# Classification of *Toona sinensis* Young Leaves Using Machine Learning and UAV-Borne Hyperspectral Imagery

**DOI:** 10.3389/fpls.2022.940327

**Published:** 2022-06-28

**Authors:** Haoran Wu, Zhaoying Song, Xiaoyun Niu, Jun Liu, Jingmin Jiang, Yanjie Li

**Affiliations:** ^1^College of Landscape Architecture and Tourism, Hebei Agricultural University, Baoding, China; ^2^Research Institute of Subtropical Forestry, Chinese Academy of Forestry, Hangzhou, China

**Keywords:** machine learning, preprocessing, classification, *Toona sinensis* young leaves, variable selection

## Abstract

Rapid and accurate distinction between young and old leaves of *Toona sinensis* in the wild is of great significance to the selection of *T. sinensis* varieties and the evaluation of relative yield. In this study, UAV hyperspectral imaging technology was used to obtain canopy hyperspectral data of biennial seedlings of different varieties of *T. sinensis* to distinguish young and old leaves. Five classification models were trained, namely Random Forest (RF), Artificial Neural Network (ANN), Decision Tree (DT), Partial Least Squares Discriminant Analysis (PLSDA), and Support Vector Machine (SVM). Raw spectra and six preprocessing methods were used to fit the best classification model. Satisfactory accuracy was obtained from all the five models using the raw spectra. The SVM model showed good performance on raw spectra and all preprocessing methods, and yielded higher accuracy, sensitivity, precision, and specificity than other models. In the end, the SVM model based on the raw spectra produced the most reliable and robust prediction results (99.62% accuracy and 99.23% sensitivity on the validation set only, and 100.00% for the rest). Three important spectral regions of 422.7~503.2, 549.2, and 646.2~687.2 nm were found to be highly correlated with the identification of young leaves of *T. sinensis*. In this study, a fast and effective method for identifying young leaves of *T. sinensis* was found, which provided a reference for the rapid identification of young leaves of *T. sinensis* in the wild.

## Introduction

*T. sinensis*, with rapid growth rate, straight trunks and beautiful texture (Liao et al., 2007), has been cultivated for more than 2000 years in China and widely used for its medicinal value and edible young leaves (Chen et al., 2017; Cao et al., 2019; Peng et al., 2019). The young leaves of *T. sinensis* are rich in nutrients such as trace elements and B vitamins, making it an ideal green food with high market value (Zhai and Granvogl, 2020). At present, the yield of young leaves is generally assessed by picking and weighing, which is time and labor consuming and not applicable to large-scale measurement. Therefore, a real-time high-throughput method is needed to identify young leaves from the old leaves and evaluate the relative yield of young leaves in the field, which is of great significance to the breeding and production of *T. sinensis*.

Although low-cost passive image sensors such as visible (RGB) have been successfully applied to the young leaf identification in some plants, higher spectral fidelity may be required, which can be provided by hyperspectral imaging (Adão et al., 2017; Wang et al., 2018; Bojie et al., 2019). Hyperspectral imaging is the imaging of the spectral bands, through which the reflectivity of the target traits in hundreds of consecutive different narrow bands can be obtained (Bohnenkamp et al., 2019). With the continuous development of optical technology, it is possible to distinguish young and old leaves with difference colors. Therefore, spectroscopic techniques have the potential to automatically measure the spatial distribution of young leaves (Chen et al., 2019). Compared with traditional methods, hyperspectral imaging technology is a faster and relatively lower-cost method to identify and assess the relative yield of young leaves, and it collects samples in a non-destructive and non-invasive manner.

With the advent of unmanned aerial vehicles (UAVs), aerial imaging has gradually become a common method for data acquisition. In recent years, advances in unmanned aerial vehicle (UAV) technology have brought new opportunities for agricultural and forestry monitoring. Compared with satellite remote sensing, UAV remote sensing has the advantages of low cost, flexibility, versatility, and providing dynamic macroscopic observations, making it popular in scientific exploration and commercial applications (Zhang and Kovacs, 2012). Due to the lower flying altitude of the UAV, higher spatial resolution can be provided (Sothe et al., 2019). UAVs can be equipped with RGB, multispectral and hyperspectral sensors. With hyperspectral sensors, which have more bands, more spectral data related to plant phenotype can be obtained, allowing better identification of young leaves. In addition, UAV hyperspectral image technology contains information in two dimensions, i.e., spectrum and space (Feng et al., 2016). The detection process is efficient and non-destructive, and can realize *in situ* detection (Adam et al., 2017). At present, UAV hyperspectral imaging technology is widely used in forestry, agriculture and other fields. For example, some studies have successfully applied UAV hyperspectral imaging technology in biomass estimation (Fu et al., 2021), crop health monitoring (Deng et al., 2020) and forest protection (Chen et al., 2018). However, research on the identification of young leaves is still limited.

At present, UAV hyperspectral imaging technology has been widely used, but there are some problems (Zhu et al., 2019a). Spectra contain a lot of information, also some interfering ones, such as noise and redundancy caused by interference from chemical or physical factors (Zhang et al., 2021). The reason for the occurrence of these interfering information is that the spectrum can be affected by nonlinearities introduced by light scatter (Rinnan et al., 2009). The interfering information may affect the accuracy and precision of spectral prediction models (Xu et al., 2008; Liu et al., 2020). However, proper preprocessing can largely eliminate these effects. Some frequently used preprocessing methods are standard normal variate (SNV), Savitzky–Golay, and first and second derivatives (Gholizadeh et al., 2016).

Traditional discriminant models are based on linear/non-linear statistical regression models that have been developed between vegetation-related parameters and spectral data (Zhu et al., 2019b). In recent years, many studies have found that machine learning methods are more suitable for estimating vegetation parameters, particularly the multi-source fusion data (Zhu et al., 2019b; Liu et al., 2021a). Machine learning has the advantage of high-performance computing and is the key to analyzing spectral information and exploring the relationship between spectral information and predicted features (Zhu et al., 2019c). In recent years, methods such as partial least squares discriminant analysis (PLS-DA), support vector machine (SVM) and random forest (RF) have been widely used in agriculture and forestry, specifically seed variety classification, crop disease detection and tree species classification (Näsi et al., 2018; Fabiyi et al., 2020; Rahman et al., 2020; Sothe et al., 2020). These classification methods have shown a powerful and promising result. However, few studies have combined machine learning methods and UAV hyperspectral to classify plant leaves with difference colors.

In this study, the classification of young and old leaves of *T. sinensis* using UAV hyperspectral imaging technology combined with different preprocessing methods and machine learning algorithms was studied. The objectives of this study were (1) testing NIR hyperspectral combined with random forest (RF), artificial neural network (ANN), decision tree (DT), partial least squares discriminant analysis (PLS-DA) and support vector machine (SVM) ability to establish an optimal model for discriminating young and old leaves of *T. sinensis*; (2) when establishing RF, ANN, DT, PLSDA and SVM models, finding out the effects of various preprocessing methods on the effects of near-infrared hyperspectral; and (3) identifying the most important wavelengths associated with the classification of young and old leaves of *T. sinensis* in model calibration.

## Materials and Methods

### Study Area and Plants

**Figure 1:**
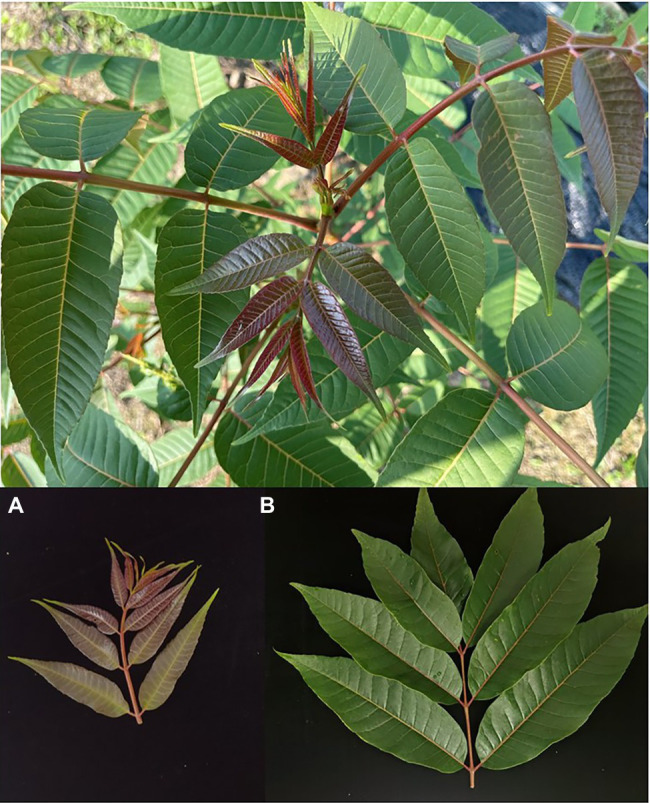
*Toona sinensis* pictures. **(A)** The young leaves pictures; **(B)** The old leaves pictures.

The experimental field (E 119°57′, N 30°03′) is located in Xinsha Island, Fuyang District, Hangzhou City, Zhejiang Province, China. *T. sinensis* seeds of different varieties from all over China were planted in the experimental field, which covers an area of 3,330 square meters. The test materials were biennial *T. sinensis* seedlings grown from these seeds. The row spacing of *T. sinensis* seedlings is 10 × 20 cm. As shown in Figure 1, the young leaves of *T. sinensis* are all fuchsia, and the young and old leaves can be clearly distinguished by the human eye.

### Hyperspectral Image Acquisition

Trials were conducted in September 2021. Image acquisition was performed using a MATRICE600 Pro UAV (Shenzhen DJI Innovation Technology Co., Ltd.) equipped with a GaiaSky-Mini2-VN (JiangSu Dualix Spectral Image Technology Co. Ltd., China) airborne hyperspectral imager with 1,392 × 1,040 pixels. The GaiaSky-mini2 airborne hyperspectral imager has a spectral range between 400 and 1,000 nm, a total of 176 spectral bands, and a spectral resolution of 3 ± 0.5 nm. The hyperspectral UAV flew at around 12:00 noon, with a flight speed of 5 m/s, and a total flight time of 4 min and 41 s. Images were collected at a flight altitude of 50 m, and the photo-taking interval was set to be equidistant at 20 m. The weather was partly cloudy during the flight. A total of 83 images were acquired.

### Hyperspectral Image Calibration and Data Acquisition

In order to reduce the influence of uneven light distribution and dark current, the hyperspectral image should be corrected for black and white (Xia et al., 2019). NIR hyperspectral images were imported into SpecView software. The lens calibration, reflectance calibration and atmospheric correction were performed using the image captured after the automatic exposure of the diffuse reflection whiteboard with the lens, the dark background captured by covering the lens, and the 50% diffuse reflection gray cloth image captured. Finally, image stitching was performed. The calibration images were imported into ENVI 5.3 software, and the region of interest (ROI) was extracted using the region of interest tool function in the software. Each pixel (i.e., ROI) contained a 176-dimensional spectral information. ROI was obtained randomly according to the obvious color difference between young and old leaves of *T. sinensis* in hyperspectral images. The old leaves obtained 1956 ROIs, and the young leaves obtained 2015 ROIs, with a total of 3,971 samples. 80% of the samples were randomly selected as the training set and 20% of the samples as the test set.

### Data Analysis

#### Spectral Preprocessing

There may be noise in the raw spectral data that can interfere with subsequent data analysis, so it is necessary to use appropriate methods to preprocess the raw spectral data (Zhang et al., 2020). In this experiment, SNV, Savitzky–Golay, first derivative, second derivative, SNV + first derivative and SNV + second derivative were used for spectral preprocessing.

#### Model Building and Performance Evaluation

RF, ANN, DT, PLSDA, and SVM algorithms were used to build models. RF is an ensemble algorithm composed of multiple decision trees proposed by Breiman (2001). The RF algorithm has the advantages of good tolerance to data noise, less manual intervention, and faster operation speed. ANN, also known as Multilayer Perceptron (MLP), consists of an input layer, a hidden layer and an output layer (Suchacz and Wesolowski, 2006). Each layer is fully connected to the previous and subsequent layers (Del Frate et al., 2007). There are a large number of nodes between network layers, and the nodes are connected by weights. ANN is shown to be a general algorithm that can be used to solve classification problems (Zhang et al., 2018). The theoretical structure of DT is a tree diagram. DT divides the population or samples into two or more homogeneous sets called sub-population based on the most important splitter in the input variable (Zhang et al., 2020). SVM is a statistical learning method based on structural risk minimization. SVM achieves data classification by finding a segmentation hyperplane between the data to maximize the separation or margin between samples of different classes (Melgani and Bruzzone, 2004). Its decision making function is determined by a small number of support vectors, enabling it to avoid the “dimension disaster” and “over-learning” problems in a sense. So it has a strong generalization ability (Zhao et al., 2020). PLSDA is a discriminative method based on Partial Least Squares Regression (PLSR; Chen et al., 2020). PLSDA establishes a linear regression between the independent variable matrix (X) and the dependent variable array (Y) to calculate the category information matrix of the test sample, and then determines the category of the samples according to the closeness of the category information matrix to the category label (Miaw et al., 2018).

All data analysis and plotting were performed in the R programming language in RStudio. Hyperspectral data preprocessing was performed using the *prospect* (Stevens and Ramirez-Lopez, 2014) software package. PLSDA, SVM, RF, DT, and ANN classification models were performed with *caret* (Kuhn et al., 2020), *e1071* (Meyer et al., 2019), *ranger* (Wright and Ziegler, 2017), *rpart* (Alfaro et al., 2013) and *nnet* (Ripley et al., 2016) packages, respectively. Model performance was evaluated by the combined accuracy, sensitivity, specificity, and precision (Liu et al., 2020). The equations are as follows:


(1) $$Accuracy=\frac{TP+TN}{TP+TN+FP+FN}$$

(2) $$Sensitivity=\frac{TP}{TP+FN}$$

(3) $$Specificity=\frac{TN}{TN+FP}$$

(4) $$Precision=\frac{TP}{TP+FP}$$

where Accuracy is the overall accuracy rate, TN is true negative, TP is true positive, FN is false negative, and FP is false positive.

#### Model Inversion

The Normalized Difference Vegetation Index (NDVI) is a band transformation, which was proposed by Kriegler et al. (1969). NDVI is effective for expressing vegetation status and quantified vegetation attributes (Huang et al., 2020). There is a difference between the NDVI of the *T. sinensis* leaves and the NDVI of the background in the photographed pictures, so the background was removed by screening NDVI. The 848.4 nm and 666.7 nm bands were selected for NDVI calculation, and the formula is as follows:


(5) $$NDVI=\frac{NIR_{848.4}-R_{666.7}}{NIR_{848.4}+R_{666.7}}$$

The data with NDVI < 0.8 in the captured pictures were excluded. The spectrum extracted from the background-removed image was inverted using the selected model.

## Results

### Average Spectrum of Original and First Derivative

**Figure 2:**
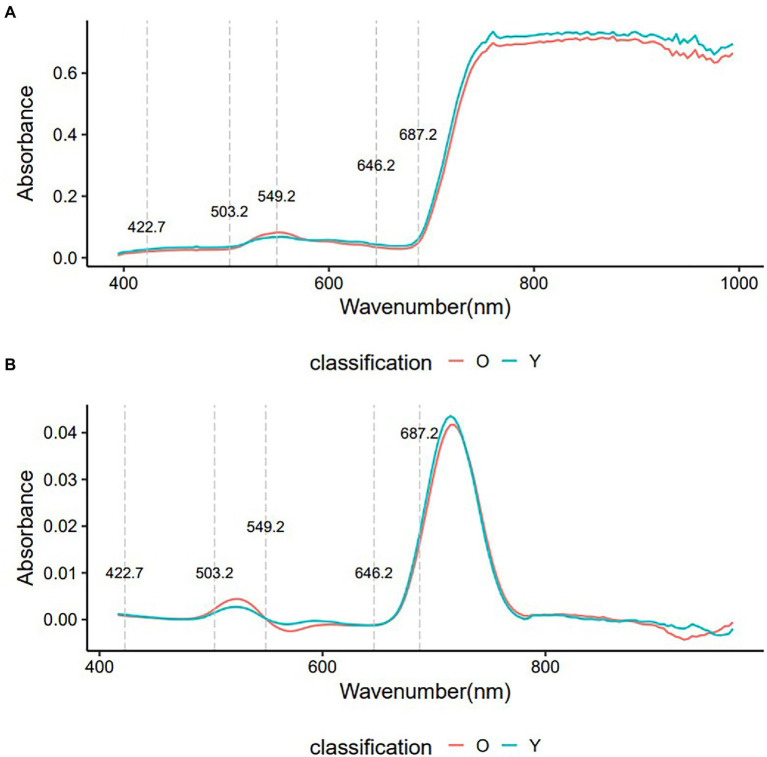
The average of VIS–NIR original spectra and first derivatives spectra of young and old leaves. O, old leaves; Y, young leaves. **(A)**: average of VIS–NIR original spectra; **(B)**: average of first derivatives spectra.

The Toona sinensis representative spectra were averaged into two groups (O: old leaves, Y: young leaves) plotted in Figure 2 based on the original and first derivative processing methods. Although the two groups had very similar shapes in the original and first derivative spectra, clear differences between the two groups of leaves were observed. The spectral differences that distinguish young and old leaves are not easily discernible in the first derivative spectra, but there are clear differences in the original spectra. Three important regions were identified: 422.7–503.2, 549.2, and 646.2–687.2 nm.

### Model Evaluation Based on Full Wavelength

**Figure 3:**
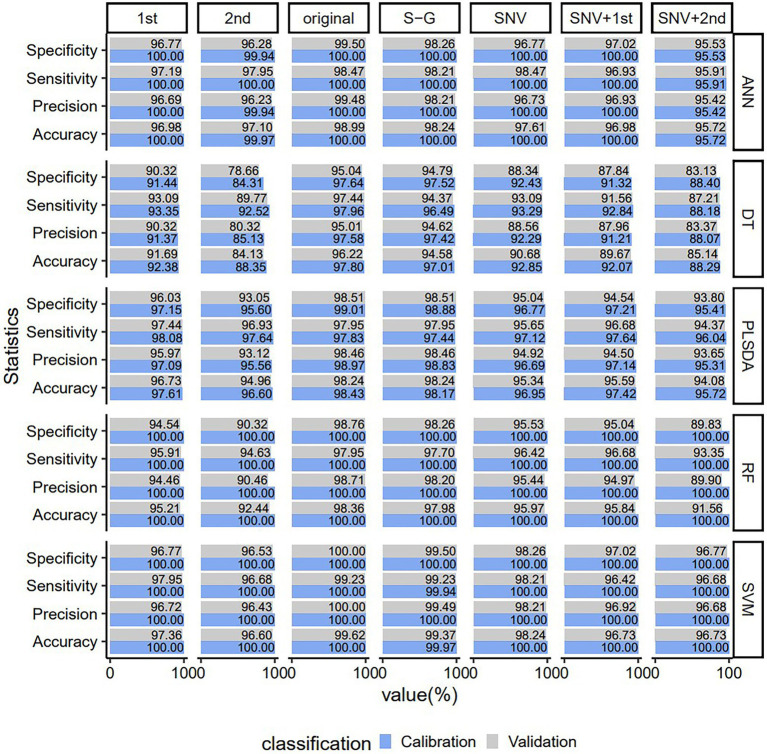
Predicting the performance of PLSDA, SVM, ANN, DT, and RF VIS–NIR classification models for young and old leaves of *Toona sinensis* based on seven VIS–NIR spectral preprocessing methods. Original, no preprocessing; S–G. Savitzky–Golay; SNV, standard normal variate; 1st Der, first derivative; 2nd Der, second derivative; SNV + 1st Der, Stsandard Normal Variate combined with first derivative; SNV + 2nd Der, Standard Normal Variate combined with second derivative.

In order to choose the best combination method for preprocessing and model, the original data and the spectral data preprocessed by SNV, Savitzky–Golay, first derivative, second derivative, SNV + first derivative and SNV + second derivative were input into DT, ANN, SVM, PLSDA, and RF models, respectively. The accuracy, sensitivity, precision, and specificity of methods based on different preprocessing methods and five model combinations are plotted in Figure 3. It can be seen that using the original spectrum had higher accuracy, sensitivity, precision, and specificity than using other preprocessings in the same model, and their values were all >95.01%. When the same preprocessing method was used, the accuracy, sensitivity, precision and specificity of the SVM model were higher than those of the other four models, and their values were all above 96.43%. Among the different preprocessing methods and five model combinations, the SVM model using raw spectra was the best, yielding the highest accuracy, sensitivity, precision and specificity (only the validation set had an accuracy value of 99.62% and a sensitivity value of 99.23%, and the rest were all 100.00%). In conclusion, in terms of classification performance, raw spectra consistently outperform other preprocessing methods. Compared with DT, ANN, PLSDA, and RF models, SVM can produce a reliable and robust classification result in most of the preprocessing spectra. Therefore, raw spectra and SVM models were chosen for future use.

### Important Variables

**Figure 4:**
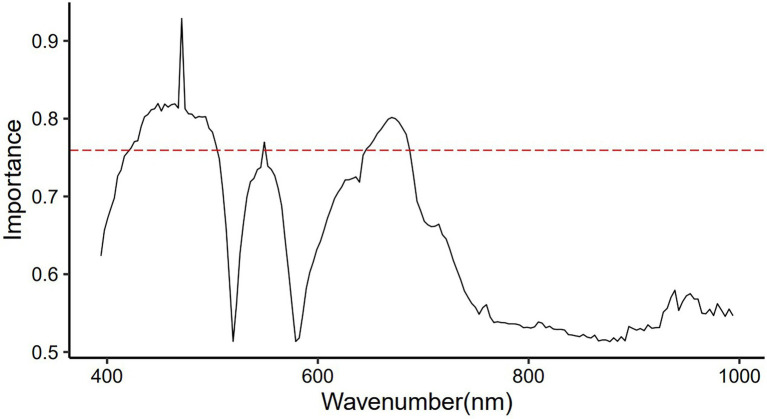
Significant values of wavelengths for distinguishing raw VIS–NIR spectra of young and old leaves of *Toona sinensis* were found by the SVM model.

Non-informative variables may affect the accuracy of the modeling. Feature wavelength selection can reduce the dimensionality of raw spectral data, retain useful information and remove redundant information. The important variable values (wavelength) of the original spectrum found by the SVM model are shown in Figure 4, and the top 40 important spectral variables whose importance value exceeded 0.75 were marked. The wavelength of 470.8 nm had a large impact on the performance of the SVM model with a value of 0.93, followed by 435.5~467.6, 474~493.5, 670.1 and 673.5 nm, which were equally important for the SVM model, with a value of 0.80 ~ 0.82. The wavelengths of 422.7 ~ 432.3, 496.7 ~ 503.2, 549.2, 646.2 ~ 676.9 and 680.3 ~ 687.2 nm were also important for the SVM model, but their values were lower, <0.80. These variables were mainly located in the three vital regions (422.7~503.2, 549.2, and 646.2~687.2 nm) found by the raw spectra (Figure 2).

### Evaluation of SVM Model Based on Full-Band and Feature-Band

**Figure 5:**
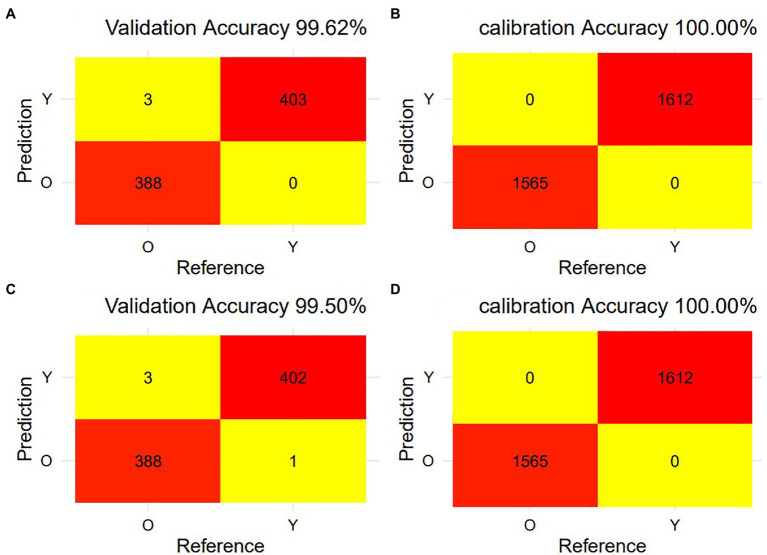
The misclassification of young and old leaves of *Toona sinensis* from calibration and validation the SVM model using raw VIS–NIR spectra and feature variable VIS spectrum. **(A)** The confusion matrix of misclassification for validation data of raw VIS–NIR spectra predicted; **(B)** The confusion matrix of misclassification for calibration data of raw VIS–NIR spectra predicted; **(C)** The confusion matrix of misclassification for validation data of feature variable VIS spectrum predicted; **(D)** The confusion matrix of misclassification for calibration data of feature variable VIS spectrum predicted.

Figure 5 shows the misclassification confusion matrix for calibration and validation of the SVM model using full-spectral data and feature-spectral data, respectively. The calibration set predicted by the SVM model based on full-spectral data had no misclassifications, and there were only three misclassifications in the validation set. The calibration set predicted by the SVM model using the feature variables had no misclassifications, and there were only four misclassifications in the validation set. The prediction accuracy of the validation set of the SVM model based on full spectrum data and feature variables was 99.62% and 99.50%, respectively. And the prediction accuracy of the calibration set based on full spectrum data and feature variables was both 100.00%. The accuracy, sensitivity, precision, and specificity (Table 1) of the SVM model based on feature variables were lower than those of the SVM model based on full spectrum data, but the values were all greater than 99.23%. Therefore, the dimensionality-reduced spectrum can also be used to accurately identify young and old leaves of *T. sinensis*. In conclusion, the vast majority of young and old leaves of *T. sinensis* can be accurately classified, which indicates that hyperspectral data combined with SVM model is a fast and non-destructive method for identifying young and old leaves.

**Table 1:** SVM model evaluation of feature variables.

Classification	Accuracy (%)	Sensitivity (%)	Specificity (%)	Precision (%)
Calibration	100.00	100.00	100.00	100.00
Validation	99.50	99.23	99.75	99.74

### Inversion of the Best Model

**Figure 6:**
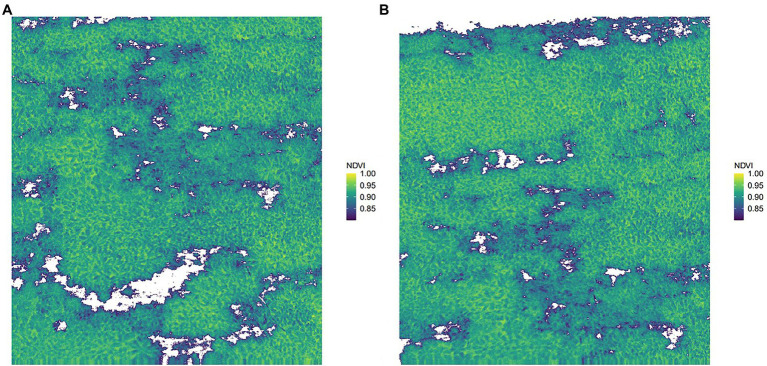
Retained graphs with NDVI > 0.8. **(A)** Original image 1 after NDVI screening; **(B)** Original image 2 after NDVI screening.

**Figure 7:**
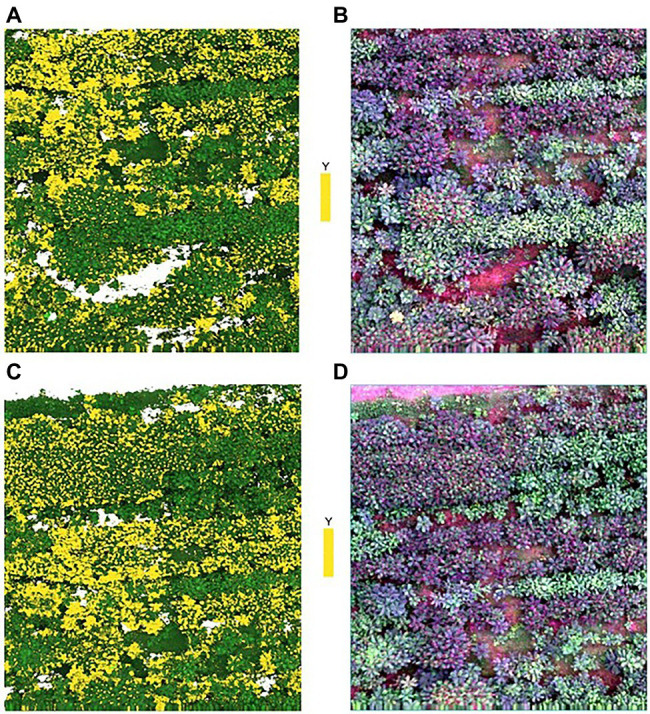
Distribution of young leaves and old leaves of *Toona sinensis* in the prediction map and the original map. Y, young leaves; **(A)** Prediction map of the original image 1; **(B)** Original image 1; **(C)** Prediction map of the original image 2; **(D)** Original image 2.

Two images (i.e., original image 1 and original image 2) were randomly selected from the captured images for prediction. Figure 6 shows the results of removing the background of the captured images after filtered by the NDVI. Comparing with the original images in Figures 7B,D, the background in the original images can be more accurately removed by the NDVI index. Then, the full spectrum-based SVM model was used for inversion, and the results are shown in Figures 7A,C. It can be seen from Figure 7 that the distribution results of young and old leaves of *T. sinensis* predicted by the SVM model are mostly the same as their real distribution in the original image. The above results show that the SVM model can be used for the identification of young and old leaves of *T. sinensis*.

## Discussion

Although the color difference between young and old leaves of *T. sinensis* is obvious, distinguishing young leaves from old leaves by human eyes is generally subjective and uncertain. Moreover, evaluating the yield of *T. sinensis* by picking is not applicable in a large area. Among the spectroscopic studies of *T. sinensis*, most studies focus on the estimation of leaf biochemistry (e.g., chlorophyll and nitrogen content) and wood chemicals (e.g., heartwood extract content; Li et al., 2020; Liu et al., 2021b). However, few studies discuss how to quickly distinguish young and old leaves of *T. sinensis,* which plays an important role in breeding and estimating relative *T. sinensis* yield and provides a technical basis for machine picking of *T. sinensis*. In this study, six preprocessing methods (SNV, Savitzky–Golay, first derivative, second derivative, SNV + 1st derivative, and SNV + 2nd derivative) and raw spectra combined with five common machine learning methods were compared using UAV hyperspectral data (RF, SVM, ANN, DT, and PLSDA) to evaluate their performance in distinguishing young and old leaves of *T. sinensis*. The results show that the combination of the original spectrum and the SVM model outperforms other combinations in the overall accuracy, providing a high-precision and high-efficiency method for the identification of young and old leaves of *T. sinensis*. The convincing results of this study show the potential of UAV hyperspectral imaging in distinguishing young and old leaves of *T. sinensis*.

Six preprocessing methods were used in this study, and the models built with raw spectra outperformed those built with other preprocessing methods. Different NIR hyperspectral preprocessing has a great impact on the output performance of the SVM model. Wu et al. (2021) used near-infrared (NIR) hyperspectral to compare the performance of different pretreatments and model combinations to distinguish waxy wheat (*Triticum aestivum* L.) and three partially waxy wheat from wild-type wheat. It was found that the SVM model based on the original spectral data had the highest classification accuracy (98.51%) for the prediction set. He et al. (2019) employed near-infrared hyperspectral imaging combined with multiple preprocessing and classification models to identify the vigor of rice seeds. It is concluded that the combined method of the original spectrum and least squares support vector machines achieves better results, with the classification accuracy over 94.00%. which are similar to the experimental results of our study. Different preprocessing methods have pros and cons. For example, smoothing can remove some noise from interfering information and improve the signal-to-noise ratio, but may also distort the original signal. The derivative can enhance spectral differences, but it tends to reduce the signal-to-noise ratio by enhancing the noise. Additionally, models obtained using derivative spectroscopy are sometimes less robust to instrumental variations (Xu et al., 2008). In practical applications, different spectral preprocessing methods should be selected according to the needs.

Different classification methods, such as PLSDA, SVM, DT, RF, and ANN, can achieve classification in different aspects. In the classification results of the five models in this study, except for the DT model using the second derivative and SNV + second derivative, the classification accuracy of the rest is above 90.00%. The classification result of the SVM model is the best. Lucas et al. (Domingos et al., 2021) used visible and near-infrared reflectance spectroscopy to effectively distinguish young and old leaves of *Hevea brasiliensis* by PLSDA classification, and the established model was robust enough. In the results of this paper, the classification accuracy of PLSDA (all above 94.08%) is also very high, and it can also be applied to the classification of young and old leaves of *T. sinensis*. Castro et al. (2018) used hyperspectral images to classify different stages of coffee rust infection. They used three classifiers (DT, SVM, and K-nearest neighbor), and finally the SVM-based classifier provided the best performance. Not only that, the rapid detection and classification of corn (*Zea mays* L.) seeds by the SVM classification model yielded a high classification accuracy of 96.46% (Tao et al., 2021). The results of these studies are consistent with the results of this study, where the SVM model shows better classification performance.

The SVM model was used in this study to reduce the high dimensionality of hyperspectral data, and three important regions were identified to distinguish young and old leaves of *T. sinensis*. The three important regions cover almost the entire visible light range: the blue/cyan edge (422.7 ~ 503.2 nm), the green peak centered at 550 nm (549.2 nm), and the red reflectance minimum (646.2 ~ 687.2 nm). The reflection in the visible band is mainly affected by the pigments in the leaves. Previous studies have identified the differences in leaf pigment content among species as an important factor for classification (Fernandes et al., 2013). Chlorophyll has the greatest effect on visible light absorption, followed by anthocyanins and carotenoids (Hennessy et al., 2020). The region of 400 ~ 700 nm has been reported to be sensitive to vegetation biochemical properties (such as canopy pigment content and nitrogen content), which is important in vegetation identification (Muchow et al., 1996; Fernandes et al., 2013; Frels et al., 2018). In particular, the band around 680 nm has become the most critical and commonly used band for crop identification (Thenkabail et al., 1999). But in this study, the importance value of the band at 470.8 nm was the largest (0.93), which may be related to the fuchsia color of its young leaves.

The filtered 40 variables were input into the SVM classifier to distinguish young and old leaves of *T. sinensis*. The SVM model using the full spectrum performs better than the SVM model using the characteristic wavelengths in our study (Figures 2, 5; Table 1). However, the difference between them is very small, and the SVM model using characteristic wavelengths can also be applied to the classification of young and old leaves. Zhu et al. (2019c) used near-infrared hyperspectral imaging to identify seven cotton (*Gossypium* spp.) seeds and the results showed that the classification model using the full spectrum performed better than the classification model using the effective wavelength. Yanqi et al. (2020) used hyperspectral imaging technology to quickly identify tea varieties. It was found that the use of characteristic spectral modeling reduced the accuracy of the discriminant model, and the use of the full spectrum combined with the SVM model to distinguish different tea varieties had an accuracy rate of up to 100%. Studies have also shown that when using hyperspectral imaging to quickly and accurately identify *Chrysanthemum* varieties, all models using full wavelengths achieved better performance than models using characteristic wavelengths (Wu et al., 2018). These are similar to the results in this paper, and the reason may be that the full hyperspectral data contains more information than the selected characteristic wavelengths. In this study, the combination method of the selected original spectrum and SVM model was applied on Toona sinensis in the experimental site, and satisfactory results were obtained (Figure 7). Therefore, it is worth considering using this combination method in future when UAV hyperspectral is applied to the classification of young and old leaves of *T. sinensis*. However, this method requires continuous validation in different species of *T. sinensis* and in different environments.

## Conclusion

This study uses UAV hyperspectral imaging technology to distinguish young and old leaves of *T. sinensis* through raw spectra and six spectral preprocessing methods combined with five machine learning models, in order to identify the optimal combination method. PLSDA, SVM, ANN, DT, and RF models all achieve satisfactory accuracy when using raw spectra. Therefore, all the five models can be used as a non-destructive and fast method to classify young and old leaves of *T. sinensis*. Furthermore, the combination of raw spectra with the SVM model is a reliable and robust classification method. This suggests that hyperspectral imaging may be a promising technique for distinguishing young and old leaves of *T. sinensis*.

## Data Availability Statement

The original contributions presented in the study are included in the article/supplementary material, further inquiries can be directed to the corresponding authors.

## Author Contributions

HW conducted the experiment and wrote the manuscript. YL designed the study, supervised experiments, and revised the manuscript. XN and JL supported the data collection and field experiments and revised the manuscript. ZS assisted in data collection and experimental operations. JJ supervised experiments and performed revisions of the manuscript. All authors contributed to the article and approved the submitted version.

## Funding

This work was funded by the Zhejiang Science and Technology Major Program on Agricultural (forest) New Variety Breeding (2021C02070-1-1).

## Conflict of Interest

The authors declare that the research was conducted in the absence of any commercial or financial relationships that could be construed as a potential conflict of interest.

## Publisher’s Note

All claims expressed in this article are solely those of the authors and do not necessarily represent those of their affiliated organizations, or those of the publisher, the editors and the reviewers. Any product that may be evaluated in this article, or claim that may be made by its manufacturer, is not guaranteed or endorsed by the publisher.
